# *Veillonella dispar* and *V. atypica* increased the growth of *Listeria monocytogenes* in liquid culture and biofilm conditions

**DOI:** 10.1371/journal.pone.0332852

**Published:** 2025-11-21

**Authors:** Fanie Shedleur-Bourguignon, William P. Thériault, Frédéric Berthiaume, Ibtissem Doghri, Jessie Longpré, Alexandre Thibodeau, Philippe Fravalo

**Affiliations:** 1 Faculté de Médecine Vétérinaire, NSERC Industrial Research Chair in Meat Safety (CRSV), Université de Montréal, Saint-Hyacinthe, Québec, Canada; 2 Vice-Dean of Research, Faculté de Médecine Vétérinaire, Université de Montréal, Saint-Hyacinthe, Québec, Canada; 3 Department of Pathology and Microbiology, Faculty of Veterinary Medicine, Université de Montréal, Saint-Hyacinthe, Québec, Canada; 4 F. Ménard, Olymel L.P., Ange-Gardien, Québec, Canada; 5 CRIPA Swine and Poultry Infectious Diseases Research Center, Faculté de Médecine Vétérinaire, Université de Montréal, Saint-Hyacinthe, Québec, Canada; 6 Chaire Agroalimentaire, USC Metabiot, Cnam Anses, Conservatoire National des Arts et Métiers (Cnam), Paris, France; Universidad Autonoma de Chihuahua, MEXICO

## Abstract

*Listeria monocytogenes* (*L. monocytogenes*) is a foodborne pathogen that causes severe illness in high-risk groups who face a mortality rate of 15% to 20% with exposure to this deadly bacterium. *L. monocytogenes* poses a significant food safety concern due to its ability to withstand the adverse conditions encountered in food production environments. Prevention of its entry into the ready-to-eat (RTE) processing environment is crucial, and consequently, preventing its establishment within the environmental microbiota of slaughterhouses—the preceding stage in the production chain—is essential. This can be a challenge because *L. monocytogenes* has the ability to create and persist in biofilms in association with microorganisms. The role of the accompanying microbiota in the survival and density of *L. monocytogenes* has been shown to range from having antagonistic to synergetic effects. The aim of the present study was to validate a positive association previously identified using bioinformatic tools between the presence of *Veillonella* spp. on conveyor belt surfaces of the cutting room of a swine slaughterhouse and the relative abundance of *L. monocytogenes*. *Veillonella dispar* (*V. dispar*) and *Veillonella atypica* (*V. atypica*) showed statistically significant positive effects on the growth and survival of the pathogen in both planktonic cultures and in biofilms tested under static and dynamic conditions. These effects of *Veillonella* appear to be mediated through compounds secreted or made available by the bacterium since contact with the supernatants of *Veillonella* cultures was sufficient to induce *L. monocytogenes* growth enhancement. This increase is primarily due to the live cell mass, suggesting that *Veillonella* acts at the *L. monocytogenes* cell population level rather than on the biofilm matrix. We believe that our results represent a step toward a better *L. monocytogenes* food safety risk assessment and could contribute to the development of better strategies against this pathogen.

## Introduction

*Listeria monocytogenes* is a Gram-positive, rod-shape foodborne pathogen and the etiological agent of human listeriosis [1]. The bacteria can cause severe illness mainly in high-risk groups such as immunocompromised and elderly individuals, in whom the invasive form of the disease can result in septicemia, meningitis, and other infections of the central nervous system. In these populations the mortality rate is between 15% and 20% [2]. Pregnant women are also at risk as the infection may lead to spontaneous abortion and fetal death [3]. The main route of human contamination is through the consumption of food contaminated by the bacteria [4].

*L. monocytogenes* poses a significant food safety concern given its ability to withstand the adverse conditions encountered in food production environments [5]. Multiple foodborne outbreaks related to *L. monocytogenes* have been linked to meat and meat products [6]. Contamination during processing has been identified by several studies as the main cause of the presence of *L. monocytogenes* in food products [7–10]. *L. monocytogenes* has the ability to attach to various surfaces and to form and persist in biofilms. Owing to the ubiquitous nature of the pathogen, prevention of its entry into the processing environment is a challenge for the food processing industry [11]. A study by Bolocan et al. has shown that a newly built meat facility can be colonized by *L. monocytogenes* in as little as four months (137 ± 7 days) after the raw materials are introduced [12]. Since the environmental microbiota will influence the bacterial contamination of meat products that are destined for the ready-to-eat (RTE) environments, the environmental microbiota of slaughterhouses is of great importance. Indeed, contamination of ready-to-eat products by *L. monocytogenes* is inherently linked to the prior introduction of the pathogen into RTE processing facilities. Raw meat cuts from upstream processing stages, such as slaughterhouses, constitute the primary route of bacterial entry into RTE production environments. Studies have attempted to identify traits that may explain why certain genotypes of *L. monocytogenes* are more commonly found in food processing facility environments as sources of cross-contamination [13,14]. The lack of individual traits allowing for the prediction of the survival of a strain of *L. monocytogenes* has given way to the idea that the subsistence of the pathogen may be due to a combination of factors [13,14], one of which being the accompanying microbiota [7].

During production at the slaughterhouse, a microbiota can survive on carcasses [15]. The microbiota can then detach and contaminate contact surfaces, such as conveyor belts, thus contaminating other food products [16]. Microorganisms present on these surfaces as well as the interactions between them can contribute to the creation of local microbial ecosystems in the production environment and to the structuring of multispecies biofilms [9]. Biofilms are known to act as reservoirs of both spoilage and pathogen bacteria in the food processing environment [17]. The involvement of biofilms in the augmentation of costs in terms of energy and time for cleaning and disinfection procedures and in the risks of food spoilage and food-related diseases has been previously highlighted [18,19]. Creating biofilms is an effective survival strategy for microorganisms since the extracellular matrix has been shown to act as a protective barrier against desiccation, heat, and antimicrobial agents [20]. Mixed biofilms can allow the colonization of transient strains and can enable the survival of weak biofilm forming bacteria which can proceed to colonize the production environment [21]. The role of the accompanying microbiota in the survival and density of *L. monocytogenes* in such microbial communities has been shown to range from having antagonistic to synergetic effects [22]. Indeed, multiple bacteria in food processing environments have been shown to have the ability to increase or decrease colonization by *L. monocytogenes* [22]. While competitive relationships can provide the potential to control the development of the pathogen in plant environments, positive interactions – described in the context of planktonic cultures and biofilms – can contribute to the persistence of *L. monocytogenes* and could represent a food safety risk [23].

Several studies conducted under planktonic co-culture conditions have demonstrated that certain bacteria such as *Pseudomonas* species as well as *Bacillus spp*. are able to enhance the growth of *L. monocytogenes* [24–28]. This positive effect has been linked to the ability of these bacteria to break down proteins in peptides and amino acids, which are then utilized by *L. monocytogenes* [22]. Another example of a positive interaction is the production of exogenous siderophores by bacteria that can stimulate the growth of *L. monocytogenes* [29]. Indeed, *L. monocytogenes* has been identified as having the ability to acquire iron associated with ferrioxamine B produced by *Streptomyces* species, thus contributing to the growth of the pathogen and increasing its chances of survival [30].

Few studies have identified positive interactions between *L. monocytogenes* and food microorganisms under mixed biofilms conditions [23,31]. Indeed, interactions that increase the biofilm formation by *L. monocytogenes* have also been reported. The protection provided by the production of extracellular polymeric substances (EPS) by bacteria around *L. monocytogenes* has been proposed as a mechanism for the pathogen’s enhanced growth in some mixed biofilms. It has also been hypothesized that a better utilization of available nutrients and an improved spatial distribution of the bacteria within the biofilm contribute to the growth of *L. monocytogenes* [22]. However, the literature on interactions between *L. monocytogenes* and the microbiota of industrial surfaces is still very sparse.

In a previous study, we identified a positive association between the genus *Veillonella* and the presence of *L. monocytogenes* on the conveyor belt surfaces of the cutting room of a swine slaughterhouse using 16S rRNA sequencing and MaAsLin biomarker analysis [32]. *Veillonella* are strictly anaerobic, non-fermentative, Gram-negative cocci that are found in the oral, respiratory, intestinal, and genito-urinary microbiota of animals, including pork [33]. *Veillonella* species, in particular *V. dispar* and *V. atypica*, are known to be early colonizers of the oral biofilm of the human mouth [34,35]. Although strains of *Veillonella* are phenotypically very similar, 12 species are recognized [33,36]. *Veillonella* are characterized by their unusual metabolic capabilities, including their use of the lactic acid produced by other bacteria from carbohydrate fermentation. *Veillonellae* are weakly adherent to hard and soft tissue surface, but they can adhere to other genera of oral bacteria, thus facilitating the formation of multispecies bacterial networks [33,36–38].

With its large data processing capacity and its ability to detect fastidious and/or nondominant bacteria, high throughput sequencing (HTS) technology has greatly expanded our understanding of the composition of complex microbial communities [7]. These bioinformatic tools and approaches allow us to establish statistical links between the presence or absence of bacteria and certain given conditions. It is however important to validate any interactions inferred from HTS using living cells in order to confirm the biological value of the results. Therefore, the aim of the present study was to validate a positive relationship between the presence of *Veillonella* spp. and the relative abundance of *L. monocytogenes* previously identified using bioinformatic tools. The concentration of the population of different strains of *Listeria monocytogenes* was first evaluated in mixed planktonic cultures with two strains of *Veillonella spp*. The spatial organization of the cell populations of *L. monocytogenes* and *Veillonella* spp. was also studied under static mixed biofilm conditions. Finally, the effect of *Veillonella* spp. supernatant on *L. monocytogenes* biofilm density was studied under static and dynamic conditions. To our knowledge, we are the first to report a positive effect *of V. dispar* and *V. atypica* on the growth of the pathogen *Listeria monocytogenes*.

## Materials and methods

### Strains

Three strains of *Listeria monocytogenes* were used in this study: LV2CP2A (L1), LV6PI6A (L2), and LV3BO5A (L3) (laboratory nomenclature). The strains were selected from a library of isolates collected on conveyor belts present in a cutting room of a swine slaughterhouse of the province of Quebec, Canada [32]. The three strains were chosen based on their ability to produce a biofilm in monoculture at 30°C and 12°C, and based on their belonging to different serotypes to allow for comparison of results with genetically distant strains. A description of the three strains of *L. monocytogenes* is presented in Table 1, though a more detailed description including of the “strong”, “moderate”, and “weak” criteria regarding biofilm formation can be found in our previous article, Shedleur-Bourguignon et al., 2022 [32]. The ATCC (American Type Culture Collection) strains *Veillonella atypica* (ATCC 17744) (V1) and *Veillonella dispar* (ATCC 17748) (V2) were chosen as they are reference strains and well characterized for their capacity to form biofilms [41,42].

**Table 1:** Description and serotype of the L1, L2, and L3 *L. monocytogenes* strains and their capacity to form biofilms.

*L. monocytogenes* strain	Serotype	Ability to form a biofilm at 30°C	Ability to form a biofilm at 12°C
L1	3C	Weak	Weak
L2	1/2b	Weak	Moderate
L3	1/2a	Strong	Strong

*L. monocytogenes* serotyped by PCR and sero-agglutination [39,40].

### Mixed planktonic culture assay

*L. monocytogenes* strains were co-cultured with *Veillonella* strains under conditions known to be favorable to both bacterial genera: at 37°C; in rich Tryptic Soy Broth (TSB) culture media (Becton Dickinson and company, Franklin Lakes, New Jersey, USA); and under anaerobic conditions using BD GasPak EZ Gas Generating Systems Incubation Containers and Thermo Scientific Oxoid AnaeroGen 3.5L Sachets (Fisher Scientific, Waltham, Massachusetts, USA). The following protocol was performed for each combination of strains; L1/V1, L1/V2, L2/V1, L2/V2, L3/V1, and L3/V2. The *Veillonella* strains were cultured on *Veillonella* agar (HiMedia Laboratories, Mumbai, India) supplemented with sodium lactate 60% (Fisher Chemical, Saint-Laurent, QC, Canada) at 37°C and under anaerobic conditions for 48 hours. Ten colonies were then used to inoculate 50 mL of TSB broth and incubated 72 hours at 37°C under anaerobic conditions. In parallel, the *L. monocytogenes* strains were cultured on blood agar (Oxoid Limited, Cheshire, England) at 37°C for 24 hours under aerobic conditions. Two colonies were used to inoculate 50 mL of TSB broth then incubated for 24 hours at 37°C under aerobic conditions. After incubation, the different *Veillonella* and *L. monocytogenes* cultures were diluted to obtain working suspensions with a concentration of 10^5^ CFU/mL. For each assay (one assay per pair of strains), four 50 mL falcons (Sarstedt Inc, Saint-Laurent, QC, Canada) containing 35 mL of TSB were inoculated as described in Table 2. The cultures and co-cultures were then incubated under agitation for 10 days at 37°C in an SIF6000R shaker (Lab companion, Massachusetts, USA) under anaerobic conditions. The optical density at 590 nm (Biowave DNA #80-3004-70, Montreal Biotech Inc., Dorval, QC, Canada) as well as the viable counts were monitored over ten days. The RAPID’*L.mono* Medium (Bio-Rad Laboratories Ltd. Montreal, QC, Canada) was used for *L. monocytogenes* counts while the *Veillonella* agar medium (HiMedia Laboratories, Mumbai, India) was used for the *Veillonella* counts. Student t-tests with a significance level of 0.05 were performed to compare the optical density and the viable counts between mono *L. monocytogenes* cultures and mixed cultures of *L. monocytogenes* and *Veillonella* at each time point. The experiments were replicated.

**Table 2:** Composition of the different cultures and co-cultures of *L. monocytogenes* and *Veillonella.*

Conditions tested in the six trials (n assays)	V1 culture (3.5mL)	V2 culture (3.5mL)	L1 culture (3.5mL)	L2 culture (3.5mL)	L3 culture (3.5mL)	TSB broth (3.5mL)
A (2)	X		X			
B (2)		X	X			
C (2)	X			X		
D (2)		X		X		
E (2)	X				X	
F (2)		X			X	
G (6)						X

The different cultures were added to 35 mL of TSB broth; X indicates when a volume was added. A-G: Each letter is associated with a specific condition.

### Supernatant production

Supernatants were produced to be used in the planktonic culture supernatant assay as well as in the biofilm trials in static and dynamic conditions. The weak/moderate biofilm-producing strains L1 and L2 were used in these assays to better visualize the effect mediated by *Veillonella* on the growth of *L. monocytogenes* strains. The L1 and L2 *L. monocytogenes* strains as well as the V1 and V2 *Veillonella* strains were cultivated on agar plates and in TSB broths as described in the mixed planktonic cultures section. After 24 hours of incubation, 3.5 mL of each 10^5^ CFU/mL working suspension was added to 12 falcons (12 falcons per tested condition (L1, L2, V1, V2) at 6 time points (T0 – T5)) containing 35 mL of fresh TSB broth. Also, 3.5 mL of uninoculated TSB was used as a negative control. Two of the 12 falcons were immediately centrifuged at 7300 g for 20 minutes at 37°C (T0) (VWR, Saint-Laurent, QC, Canada), filtered with 0.2uM filters (Sarstedt Filtropur 0.2, Montreal, QC, Canada), and then separated into 5 mL aliquots and stored at −80°C (T0). The remaining falcons were incubated under agitation at 37°C under anaerobic conditions for 24 hours (T1), 48 hours (T2), 72 hours (T3), 96 hours (T4), or 144 hours (T5). Two falcons per combination were collected for each time (T1–T5) from the incubator and the same procedure (centrifugation, filtration, and freezing) was performed. Each experiment involving the use of supernatants was performed, including technical and biological replicates, using the supernatants produced during the same manipulation to minimize potential variations between supernatant batches.

### Evaluation of supernatant activity in planktonic cultures

Two mL of the different supernatants (V1 – T0, T1, T2, T3, T4, or T5 supernatants (SV1) and V2 – T0, T1, T2, T3, T4, or T5 supernatants (SV2)) were added to falcons containing 1.6 mL of fresh TSB broth and 0.4 mL of a culture of L1 or L2 at a concentration of 10^5^ CFU/mL. Also, as experimental controls, 2 mL of the L1 – T0, T1, T2, T3, T4, or T5 supernatants (SL1) and 2 ml of the L2 – T0, T1, T2, T3, T4, or T5 supernatants (SL2) were added, respectively, to falcons containing 1.6 mL of TSB broth and 0.4 mL of a culture of L1 or L2 (at a concentration of 10^5^ CFU/mL). The solutions were incubated at 12°C under agitation for 24 or 48 hours under aerobic conditions. Viable counts of *L. monocytogenes* suspensions representing the different combinations of *L. monocytogenes* strains, supernatants, and contact times were conducted with the RAPID’*L. mono* Medium for an incubation time of 24 hours at 37°C under aerobic conditions.

### Evaluation of supernatant activity on monospecies biofilms in static conditions

**Fig 1:**
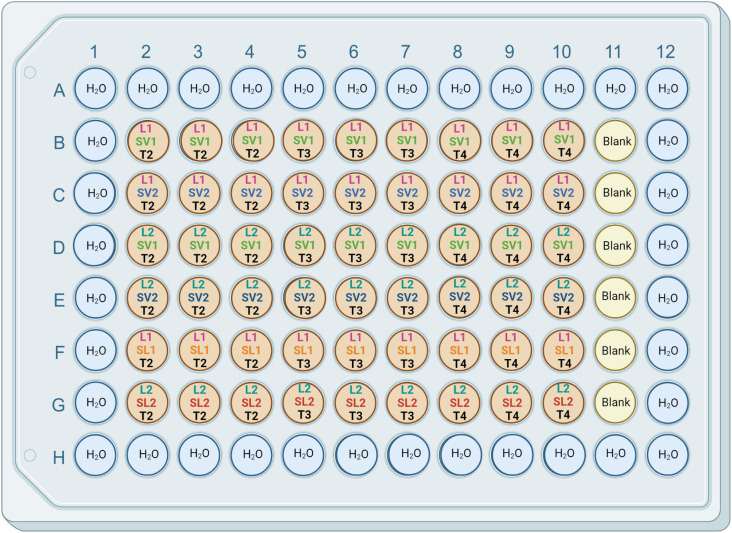
Static Biofilms Assay Plates plan. The wells of 96-well plates were inoculated with a mix of L1 or L2 *L. monocytogenes* strain cultures, TSB broth, and *Veillonella* (V1 or V2) or *L. monocytogenes* (L1 or L2) strain supernatants (T2, T3, or T4). Created with BioRender.com.

The wells of 96-well plates (Corning Incorporate, Corning, New York, USA) were inoculated with 100 µL of a mix of 0.4 mL of L1 or L2 culture, 1.6 mL of fresh TSB broth, and 2 mL of V1, V2, L1 or L2 (T2, T3, or T4) supernatants. Each combination was tested in triplicate (see the plate plan below) (Fig 1). The plates were incubated at 12°C for 10 days under humid aerobic conditions. Humid conditions were maintained by inoculating the peripheral wells of the plates with sterile water. It should also be noted that wells B11 to G11 were inoculated with TSB and used as blanks. Crystal violet (1%, filtered at 0.45 µM) (Fisher Scientific, Waltham, Massachusetts, USA) colorations were performed at days four, six, seven, and ten. Briefly, the TSB media was removed and three washes with 150 µL of sterile water were then performed. After each wash, the wells were emptied. A drying time of 10 min at room temperature occurred after the third wash. Next, 50 µL of a crystal violet solution was added to each well and an incubation time of 30 min at room temperature was carried out. Three washes with 150 µL of sterile water were again performed and the wells were emptied after each wash. A drying time of 10 min at room temperature again occured. Finally, 200 µL of 90% ethanol was added to each well 30 min before the reading of the absorbance at 595 nm (Montreal Biochrom Inc., model EZ read 400, Dorval, QC, Canada). The assays were repeated independently on two different days.

### Biofilm assay under dynamic conditions

#### Biofilm formation.

Biofilm formation under dynamic conditions was performed using the BioFlux 200 system with 48-well plates (Fluxion biosciences, South San Francisco, California, USA). The protocol developed by Benoit et al. (2010), Tremblay et al. (2015), and adapted for *L. monocytogenes* by Cherifi et al. (2017) was used with several modifications as described below [43–45]. The biofilm of the *L. monocytogenes* L2 strain was tested to compare the impact of three conditions: exposure to the (T2) 48-hour supernatant of the L2 strain during the static adherence phase (4 hours) and the dynamic flow phase (20 hours) (Condition A), exposure to the (T2) 48-hour supernatant of the V2 strain during the static adherence phase (4 hours) and the dynamic flow phase (20 hours) (Condition B), and finally exposure to the (T2) 48-hour supernatant of L2 strain during the static adherence phase (4 hours) followed by exposure to the (T2) 48-hour supernatant of the V2 strain during the dynamic flow phase (20 hours) (Condition C). The L2 strain was selected among the three strains of the study for its characteristics of interest in food microbiology, specifically its serotype 1/2b designation and its ability to form a biofilm at 12 °C. For all conditions, L2 strains were cultured on blood agar at 37°C under aerobic conditions overnight. Ten mL of TSB broth was then inoculated with one colony and incubated for 16hrs at 37°C under agitation (180 rpm), and 1.5 mL of the overnight culture was centrifuged at 4000g at 4°C (Symphony 2417R, VWR, Radnor, Pennsylvania, USA) for one minute and resuspended in 1 mL of TSB broth preheated at 30°C. The optical density was measured at 600 nm (OD600 = 1.0) to validate the growth of the strain. Afterwards, 100 µL of a prewarmed 1/10 TSB diluted broth was added to the output well of a 48-well plate and was injected at 10 dyne/cm^2^ for 10 seconds in order to precoat the growth chamber. The excess liquid in the outlet well was then removed. A drop of the broth was left at the bottom of the well and then 100 µL of the bacterial culture solution diluted at 50% with the appropriate supernatant was added to the outlet well. A 100uL of prewarmed TSB/10 broth was added to the inlet well to avoid the movement of liquid due to gravity. The bacterial culture was injected at 0.5 dyne/cm^2^ for 25 seconds. An incubation time of four hours at 30°C without flow was then applied to allow for cell adhesion. After four hours, 1.25 mL of the appropriate prewarmed supernatant was added to the input well and injected at 0.5 dyne/cm^2^ for 20 hours at 30°C. Biofilm growth was monitored by taking pictures before the adhesion time (T0), after the four-hour adhesion time (T4), and following the 20-hour incubation time (subjected to shear stress) (T24) using an inverted fluorescence microscope (Olympus CKX41) equipped with 10X and 40X objectives and a digital camera (Retiga EXi; QImaging). The three conditions were each repeated independently on different days.

#### Biofilm staining.

Biofilms were stained under dynamic conditions with SYTO 9, that allows for the visualization of nucleic acid in green, and with Propidium iodide, which colors the damaged or dead cells in red (Molecular probes, Eugene, OR, USA). Three µL of SYTO 9 and Propidium iodide were added to 1 mL of prewarmed (30°C) filtered sterile water. After 20 hours of growth under shear force, the flow was stopped and the remaining liquid volumes were removed from the inlet and outlet wells. Afterwards, 100 µL of the lived/dead dye solution was added to the inlet well and injected for 20 min at 0.5 dyne/cm^2^. Excess dye was removed from the inlet well and the well was washed with 250 µL of sterile filtered water to remove residual dye. Two hundred fifty µL of TSB/10 broth was added to the inlet well and injected for 20 min at 0.5 dyne/cm^2^. The flow was then stopped, the excess liquid was removed from the input and output wells, and 100 µL of TSB/10 broth was added to the two wells in order to balance them.

#### Biofilm visualization.

Image acquisition of biofilms stained with live/dead fluorescent coloration was performed with a Confocal Laser Scanning Microscope (CLSM, Olympus FV1000 IX81) equipped with a 40X objective. The green fluorescence of SYTO 9 was excited at 488 nm, and the fluorescence emitted was collected between 500 and 555 nm. The red fluorescence of Propidium iodide was excited at 543 nm, and the fluorescent emission was collected between 555 and 625 nm. Three dimensional images (3D) were constructed for each condition using 13–25 image layers separated from each other by 1.47 µm from the bottom to the surface of the biofilms. ISO images of the live and dead biomass were created, which allowed for the calculation of the volume of each biofilm. Approximations of the total biomass was obtained by summing the dead and living biomass as PI penetration in damaged cells causes a reduction in SYTO 9 fluorescence. The biovolumes were compared using Student t-test (with a significance level of *p* < 0.05). The 3D images, the ISO images, and the biovolume calculations were performed using the Image-Pro 3D Suite version 6.1 (Media Cybernetics, Inc., Bethesda, MD, USA).

### Mixed biofilm assay under static conditions

The L1 *L. monocytogenes* and V2 *Veillonella* strains were separately cultured on agar plates and TSB broth as described in the planktonic culture section. Three conditions were tested. The first suspension consisted of 3.5 mL of the *L. monocytogenes* culture at a concentration of 10^7^ CFU/mL added to 31.5 mL of TSB/10 broth (L). The second one consisted of 3.5 mL of the *Veillonella* culture at a concentration of 10^7^CFU/mL and 31.5 mL of TSB/10 broth (V). And the third consisted of 3.5 mL of the *L. monocytogenes* culture at a concentration of 10^7^ CFU/mL, 3.5 mL of the *Veillonella* culture at a concentration of 10^7^ CFU/mL, and 28 mL of TSB/10 (M). One hundred µL of each solution was distributed into the wells of a 96-well plate. The plate was incubated for 48 hours at 37°C under humid and anaerobic conditions. The biofilms were then stained using the ViaGram^TM^ Red^+^ Bacterial Gram Stain and Viability kit (Invitrogen, Oregon, USA). The protocol was modified to adapt the staining to biofilms. Briefly, the TSB was removed by turning the plate over. A wash was then performed with 150 µL of BSA-saline (Sigma-Aldrich, Saint-Louis, Missouri, USA, Fisher Scientific, Waltham, Massachusetts, USA) and the medium was removed again by inverting the plate. Afterwards, 52.5 µL of Texas Red-X conjugate mixture (50 µL of BSA-saline and 2.5 µL of Texas Red-X conjugate) was added to the L and M wells. The Texas Red-X conjugate selectively binds to the surface of gram-positive bacteria and strains them fluorescent red. A waiting time of 15 min at room temperature was observed. Two washes were performed with 150 µL of BSA-saline, and the liquid was removed again by inverting the plate. After that, 52.5 µL of the DAPI/SYTOX Green mixture (2.5 µL of DAPI solution, 2.5 µL of SYTOX Green solution, and 50 µL of BSA-saline) was added to the V and M wells. An incubation time of 20 min at room temperature was observed, and the liquid was removed by inverting the plate. Bacteria with intact cell membranes stain fluorescent blue (DAPI) whereas bacteria with damaged membranes stain fluorescent green (SYTOX Green). Two washes were performed with 150 µL of BSA-saline, and the liquid was removed again by inverting the plate. Fifty µL of BSA-saline was added to each well. Image acquisition of biofilms stained with ViaGram^TM^ Red^+^ Bacterial Gram Stain and Viability kit fluorescent coloration was performed with a Confocal Laser Scanning Microscope (CLSM, Olympus FV1000 IX81) equipped with a 40X objective. The blue fluorescence of DAPI was excited at 405 nm and the fluorescent emission was collected between 425–475 nm; the green fluorescence of STYOX Green was excited at 488 nm and the fluorescent emission was collected between 500–540 nm; and the red fluorescence of Texas Red was excited at 543 nm and the fluorescent emission was collected between 560–660 nm. 3D images were constructed for each condition using 20 image layers for the V, M, and L biofilms. The conditions were each repeated independently twice.

## Results

### Absorbance and viable counts of *L. monocytogenes* in mixed planktonic cultures with *Veillonella*

**Fig 2:**
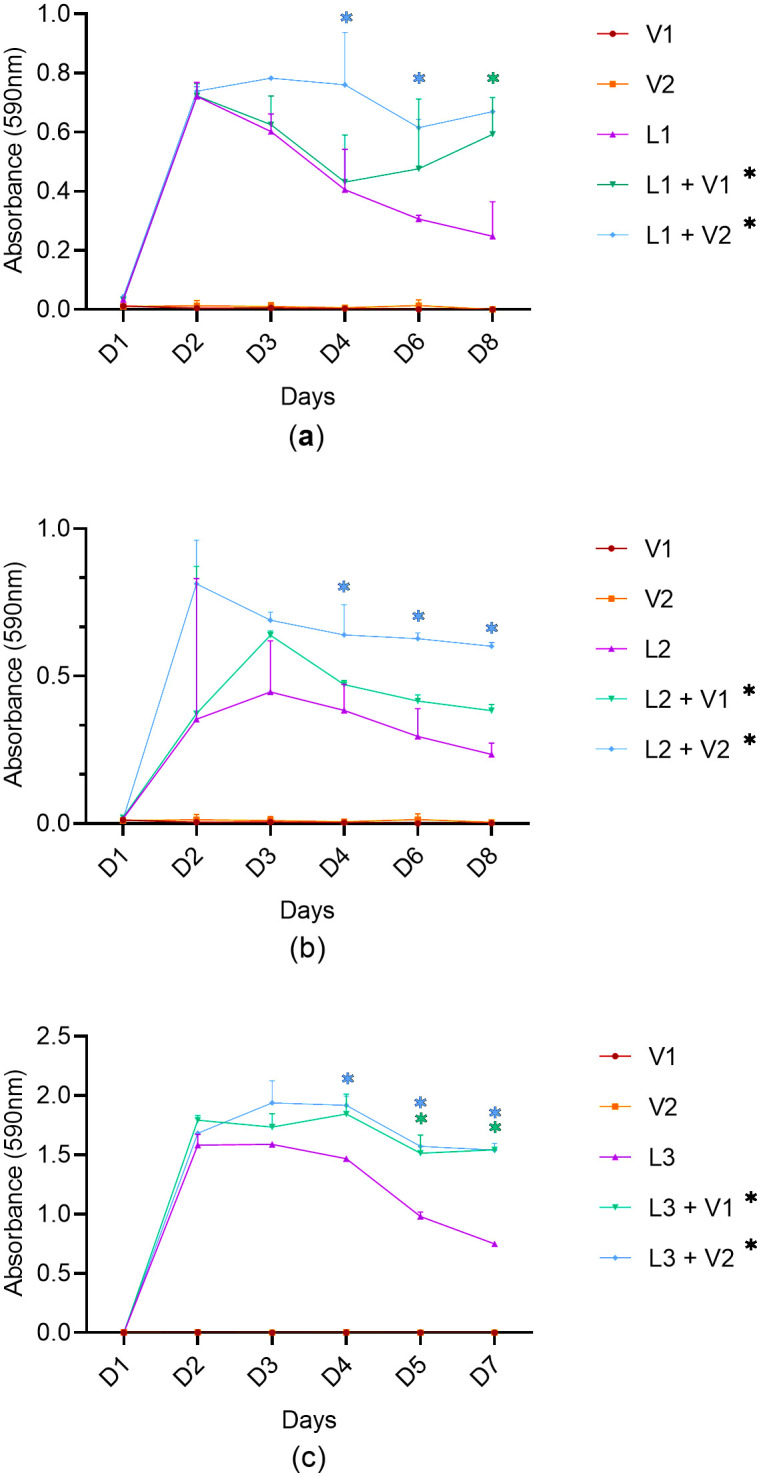
Absorbance measurements of mono *L. monocytogenes* cultures and mixed cultures of *L. monocytogenes* and *Veillonella* at 37°C. a) Mono- and co-cultures involving L1; b) Mono- and co-cultures involving L2; c) Mono- and co-cultures involving L3. In red: monoculture of V1; in orange: monoculture of V2; in purple: monoculture L1, L2, or L3; in green: co-culture of L1, L2, or L3 with V1; in blue: co-culture of *L. monocytogenes* L1, L2, or L3 with V2. *Indicates a statistically significant result.

**Fig 3:**
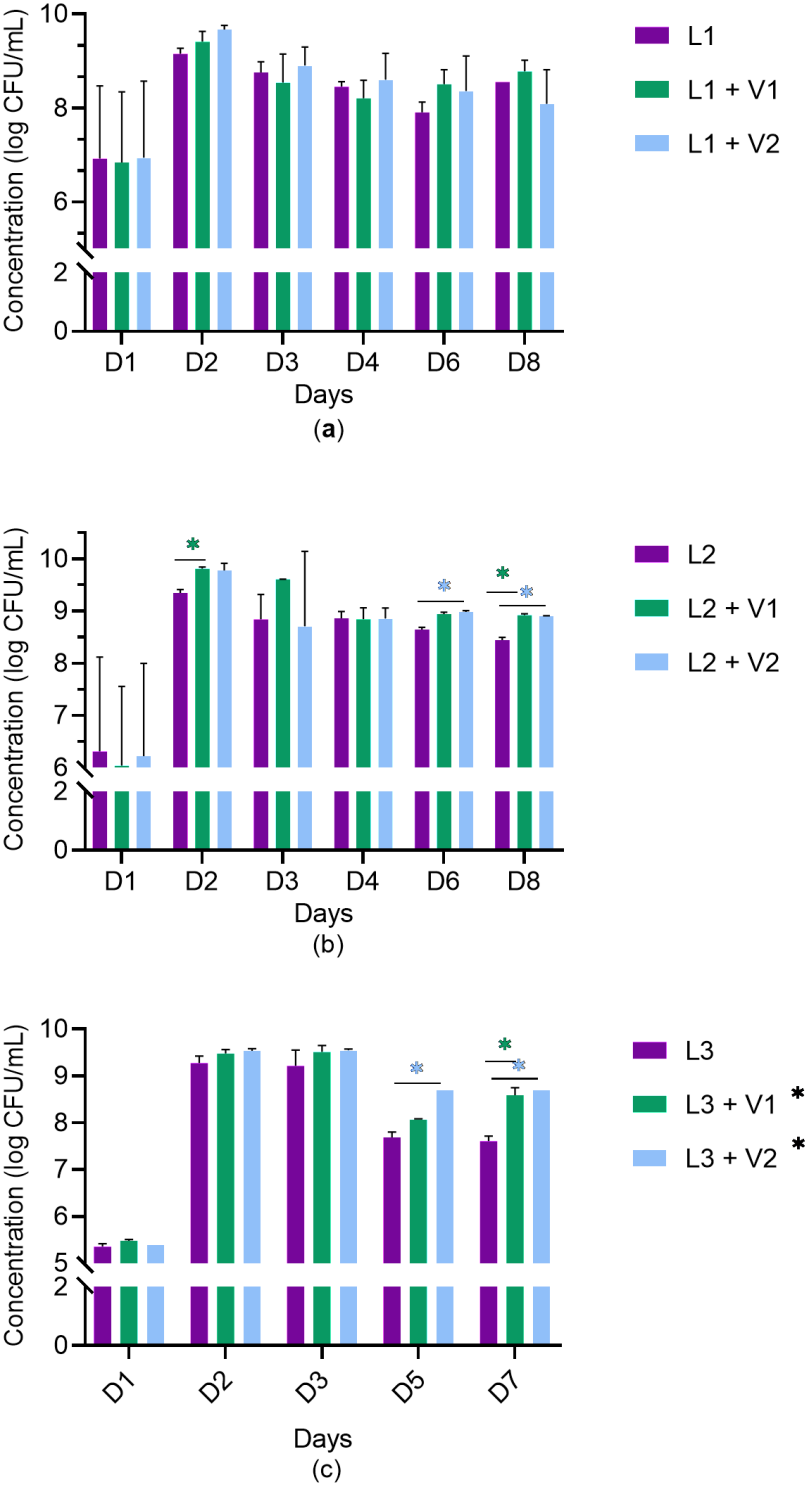
Viable counts of *L. monocytogenes* in monocultures and mixed cultures with *Veillonella* at 37°C. a) mono- and co-cultures involving L1; b) mono- and co-cultures involving L2 and c) mono- and co-cultures involving L3. In purple: monoculture of *L. monocytogenes* (L1, L2, or L3); in green: co-culture of *L. monocytogenes* (L1, L2, or L3) with *Veillonella* strain 17744 (V1); in blue: co-culture of *L. monocytogenes* (L1, L2, or L3) with *Veillonella* strain 17748 (V2). *Indicates a statistically significant result.

*Listeria monocytogenes* strains L1, L2, or L3 were co-cultured with *Veillonella* strains V1 or V2. Two replicates were performed. Figs 2 and 3 respectively show the absorbance measurements and the *L. monocytogenes* viable counts in mono- or co-cultures. The mono-cultures of *Veillonella* strains showed almost zero absorbance measurements, which did not differ from the non-inoculated control. Higher absorbance measurements and viable counts were observed in the mixed cultures of *L. monocytogenes* and *Veillonella* compared to the *L. monocytogenes* monocultures alone. However, due to heterogeneity among the trials, the ability to obtain significant differences in the Student t-tests on certain days (p ≤ 0.05) was limited.

### Supernatant activity evaluation in planktonic cultures

**Fig 4:**
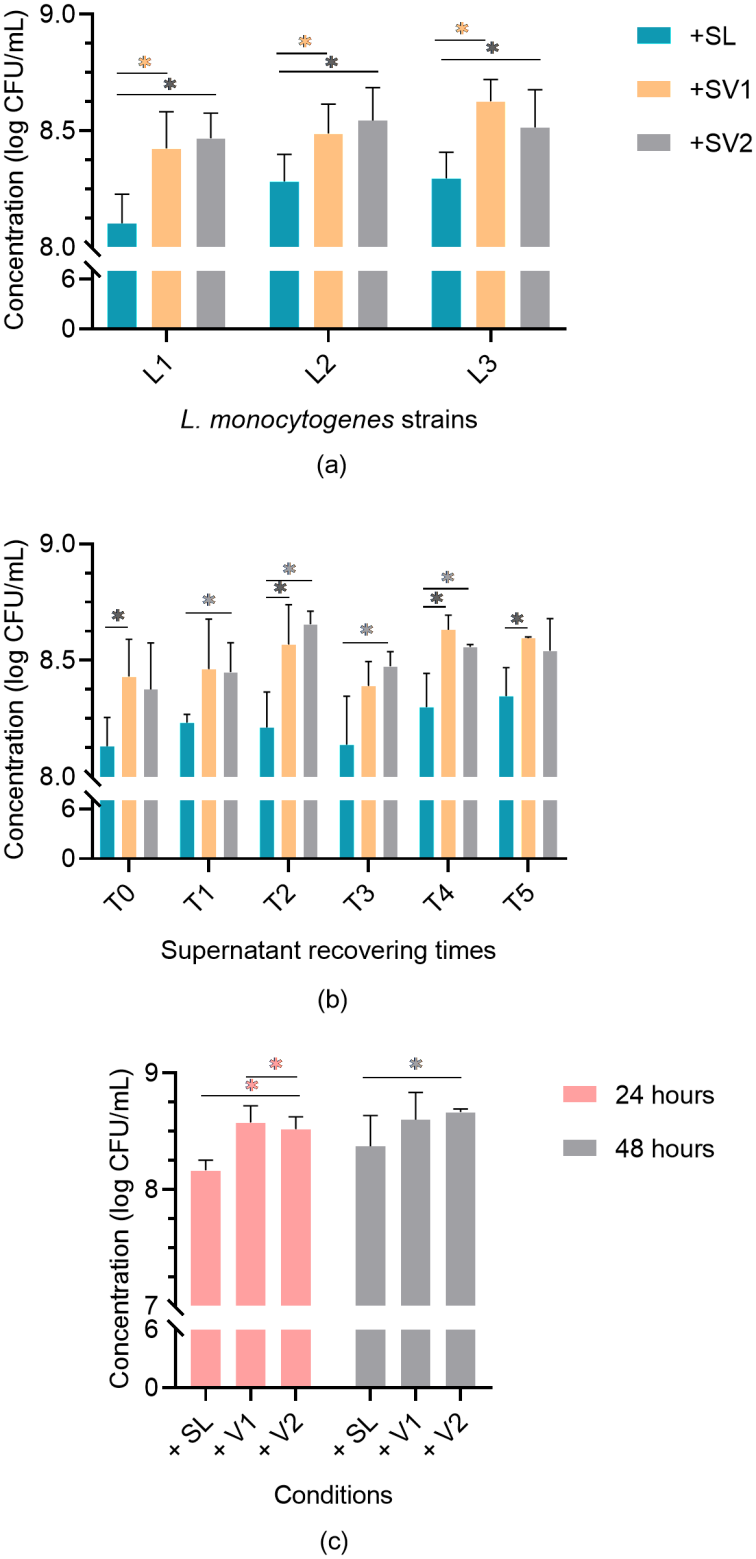
L. *monocytogenes* cultures viable cell counts when exposed to different supernatants at 12°C. a) Mean viable counts (all harvesting times combined) of the three *L. monocytogenes* cultures (L1, L2, and L3) exposed for 24h to their own supernatants (SL1, SL2, or SL3) or to the supernatant of *Veillonella* (SV1 or SV2); b) Impact of supernatant harvesting times (T0, T1, T2, T3, T4, or T5) on mean viable counts of *L. monocytogenes* cultures (L1, L2 and L3) after 24 hours of contact; c) Impact of the contact time (24 or 48 hours) between the *L. monocytogenes* cultures (L1, L2 and L3) and the different supernatants.

Viable counts of L1, L2, and L3 *L. monocytogenes* in culture with their own supernatants (SL1,SL2 or SL3) or with *Veillonella* supernatants (SV1 or SV2) were carried out in order to determine if the presence of *Veillonella* cells is necessary for *L. monocytogenes* growth enhancement (Fig 4). The impact of supernatant collection time (T0, T1, T2, T3, T4, or T5) as well as the impact of contact time (24 or 48 hours) between supernatants and *L. monocytogenes* cultures are also presented at Fig 4. All three strains of *L. monocytogenes* (L1, L2, or L3) showed significantly higher viable counts when exposed to *Veillonella* supernatants than when exposed to their own supernatants (Student t-tests with a significance level of 0.05). The T2 and T4 supernatant harvesting times showed the highest differences in *L. monocytogenes* viable counts between conditions where *L. monocytogenes* was exposed to its own supernatant or exposed to *Veillonella* supernatants (Student t-tests with a significance level of 0.05). *L. monocytogenes* cultures exposed to *Veillonella* supernatants for 24 hours showed significantly higher viable cell counts compared to when exposed to their own supernatants for 24 hours. This difference persisted after 48 hours of contact for the L + SV2 combination but not for L + SV1.

### Biofilm assay using supernatants under static conditions

**Fig 5:**
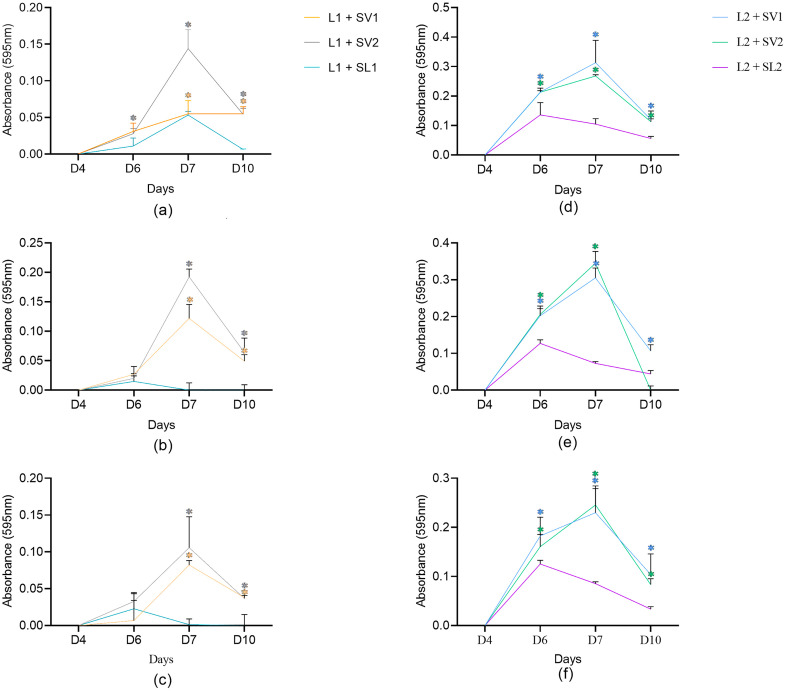
*Listeria monocytogenes* biofilm biovolume (crystal violet) at 12°C under static conditions. a) Absorbance measurements of L1 exposed to different T2 supernatants; b) Absorbance measurements of L1 exposed to different T3 supernatants; c) Absorbance measurements of L1 exposed to different T4 supernatants; d) Absorbance measurements of L2 exposed to different T2 supernatants; e) Absorbance measurements of L2 exposed to different T3 supernatants; f) Absorbance measurements of L2 exposed to different T4 supernatants. L1+SV1 in light blue; L1+SV2 in medium blue; L2+SV1 in light green; L2+SV2 in medium green; L1+SV1 in dark blue; and L2+SL2 in dark green. *Indicates a statistically significant result.

*Listeria monocytogenes* strains L1 and L2 biofilms were grown for 10 days under static conditions in TSB broth mixed with 2 mL of different supernatants: the T2, T3, or T4 supernatant of a culture of *L. monocytogenes* (SL1 or SL2); the T2, T3, or T4 supernatant culture of V1 (SV1); or the T2, T3, or T4 supernatant culture of V2 (SV2). The absorbance measurements of the different *L. monocytogenes* strain/supernatant combinations after crystal violet staining are presented in Fig 5. *L. monocytogenes* biofilms exposed to *Veillonella* supernatants (SV1 and SV2) showed significantly higher biomasses compared to the biofilms exposed to *L. monocytogenes* supernatants according to Student t-tests (р ≤ 0.05). The only exceptions were the L1 biofilms grown in contact with T3 and T4 SV1 and SV2 after six days of incubation as well as the L2 biofilm in contact with T3 SV2 after ten days of incubation.

### Biofilm assay using supernatants under dynamic conditions

**Fig 6:**
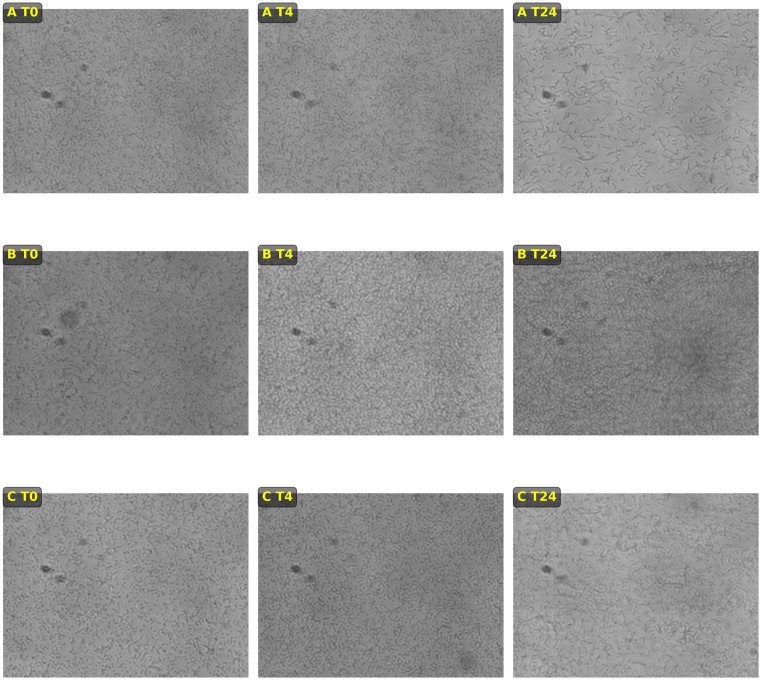
*Listeria monocytogenes* biofilm growth under dynamic conditions subjected to the contact of different supernatants. A – Exposure to the 48-hour L2 (SL2) supernatant during the adhesion and flow phases; B – Exposure to the 48-hour V2 (SV2) supernatant during the adhesion and flow phases; C – Exposure to the 48-hour L2 (SL2) supernatant during the adhesion phase (4 hours) followed by exposure to the 48-hour V2 supernatant (SV2) during the flow phase (20 hours). T0 corresponds to the time at which the cells were put in the growth chamber; T4 is the time following the four hours of adhesion; and T20 is the time following the 20-hour flow phase. Created with BioRender.com.

*Listeria monocytogenes* strain L2 biofilms were grown under dynamic conditions and exposed to different supernatants: the 48-hour L2 (SL2) supernatant during the adhesion and flow phases (Condition A), the 48-hour V2 (SV2) supernatant during the adhesion and flow phases (Condition B), or the 48-hour L2 (SL2) supernatant during the adhesion phase (4 hours) followed by exposure to the 48-hour V2 supernatant (SV2) during the flow phase (20 hours) (Condition C). Biofilm production was monitored by taking pictures before the adhesion time (T0), after the four-hour adhesion time (T4), and following the 20-hour incubation time subjected to shear stress (T24) using an inverted fluorescence microscope (Fig 6). All the replicates exposed to Condition B showed a visible increase in the growth and attachment of *L. monocytogenes* cells after four hours of contact (T4) compared to the biofilms exposed to Condition A. This difference seems to increase after 24 hours of contact (20 hours of flow). Indeed, the biofilms submitted to 24 hours of contact with the SV2 supernatant (20 hours of flow) appear to be stronger and denser than the biofilms exposed to Condition A. The biofilms exposed to Condition C showed intermediate production and fixation levels (between conditions A and B) after the fixation phase (T4) and the flow phase (T24).

**Fig 7:**
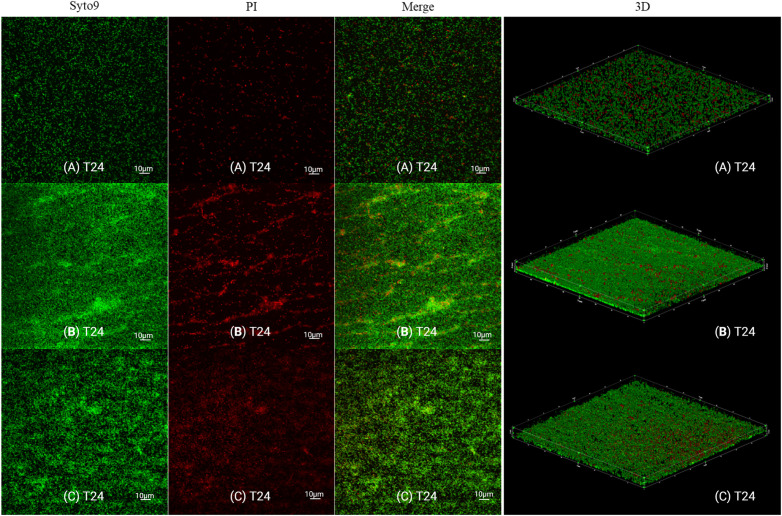
Composition of dead and live cells in the biofilms of *L. monocytogenes* strain L2 exposed to the contact of different supernatants. SYTO 9 – Individual visualization of live population; PI – Individual visualization of the dead population; Merge – Merger of the live and dead populations (total biomass); 3D – Three dimensional images constructed using 13 to 25 image layers separated from each other by 1.47 µm from the bottom to the surface of the biofilms. A – Exposure to the 48-hour L2 (SL2) supernatant during the adhesion and flow phases; B – Exposure to the 48-hour V2 (SV2) supernatant after 48 hours during the adhesion and flow phases; C – Exposure to the 48-hour L2 (SL2) supernatant during the adhesion phase (4 hours) followed by exposure to the 48-hour V2 supernatant (SV2) during the flow phase (20 hours). The 3D images were performed using the Image-Pro 3D Suite, version 6.1. Created with BioRender.com.

**Fig 8:**
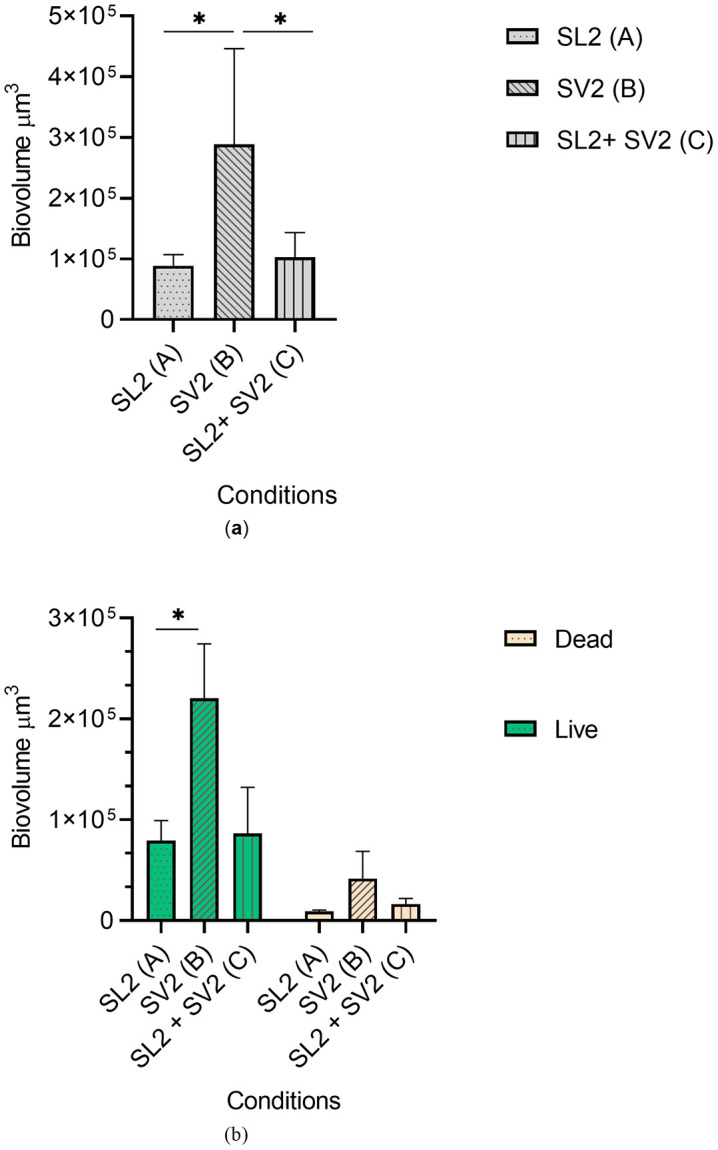
Biovolume calculations of biofilms formed in microfluidic conditions by *Listeria monocytogenes* L2 exposed to the contact of different supernatants. a) Total biovolumes of biofilms; b) Biovolumes of live and dead biomass in each biofilm type. SL2 – Exposure to the 48-hour L2 *L. monocytogenes* strain supernatant; SV2 – Exposure to the 48-hour *Veillonella* V2 strain supernatant; SL2 + SV2 – Exposure to the 48-hour *L. monocytogenes* L2 supernatant followed by exposure to the 48-hour *Veillonella* V2. supernatant. *р < 0.05.

After 24 hours, the biofilms were stained with live/dead dye and the image acquisitions were performed with a Confocal Laser Scanning Microscope (Fig 7). Similar observations were made to the ones obtained with the inverted fluorescence microscope. The biovolumes of live, dead, and total biofilm biomass were extracted from 3D images. The *L. monocytogenes* biofilms exposed to Condition A showed an average biovolume of 88,418 µm^3^ made of 90% live cell mass and 10% dead biomass. The biofilms exposed to Condition B showed for their part an average biovolume of 288.10^3^µm^3^ made of 85% and 15% of live and dead cells, respectively. Finally, the biofilms subjected to Condition C showed an average biovolume of 102.10^3^µm^3^ composed of 84% live cell mass and 16% dead biomass. Biovolumes were compared using Student t-tests (Fig 8). *L. monocytogenes* biofilms exposed to Condition B showed a significantly higher total biomass than the biomass obtained for the *L. monocytogenes* biofilm exposed to Condition A (*p* = 0.042) and to Condition C (*p* = 0.049). When the dead and live live cell mass of the biofilms exposed to the different supernatants (conditions A, B, and C) were compared, only the biovolumes of the live cells of the *L. monocytogenes* biofilm exposed to Condition B showed a significatively higher biomass than the live cell mass of the biofilm exposed to Condition A (*p* = 0.03). All other biovolume comparisons were non-significant, although biofilms formed under Condition B were found to have consistently higher biovolumes than the biofilms exposed to Condition A (3.3 times more cells) and to Condition C (2.8 times more cells). The biofilms exposed to Condition C showed a total mean biovolume only slightly higher (1.2 times) than the biofilms exposed to Condition A. However, it should be noted that the mean dead biomass of the biofilms exposed to Condition C was on average 1.8 times higher than the biofilm mean dead biomass exposed to Condition A. Considering these results, the *L. monocytogenes* biofilms exposed to the supernatant of *Veillonella* (SV2) for 24 hours showed a visible increase in the growth and attachment of *L. monocytogenes* cells (L2) compared to *L. monocytogenes* biofilms exposed to their own supernatants.

### Mixed biofilms under static conditions

**Fig 9:**
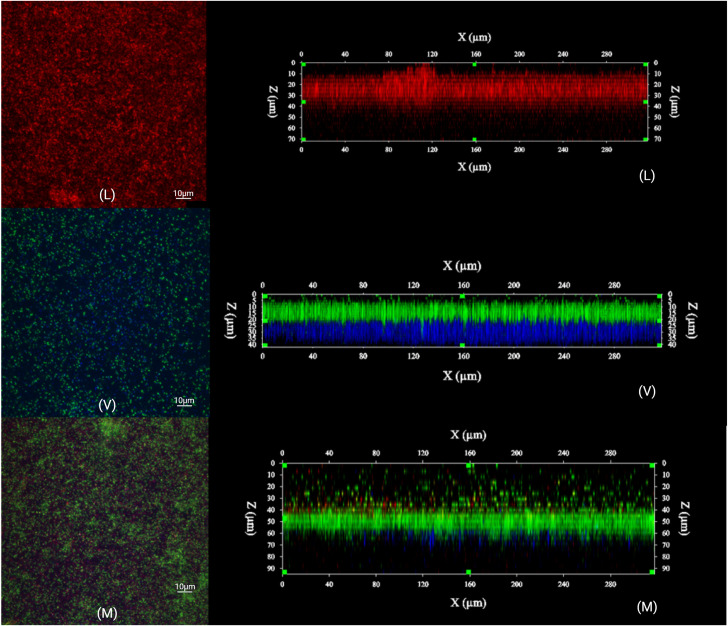
CLSM images of the organization of biofilms of *Listeria monocytogenes* L2 and *Veillonella* V2 after 48 hours at 37°C under anaerobic conditions. L – *Listeria monocytogenes* L2 mono-species biofilm; V – *Veillonella* V2 mono-species biofilm; M – dual-species biofilm of *Listeria monocytogenes* L2 and *Veillonella* V2. Blue indicates live bacteria, green indicates dead bacteria, and red indicates *Listeria monocytogenes* (Gram+). The images were performed using the Image-Pro 3D Suite, version 6.1. Created with BioRender.com.

The visualization of the mono- and dual-species biofilms of L2 and V2 was performed. The cells were stained using the *ViamGram*^TM^ Bacterial Gram Stain and Viability kit and the image acquisitions were performed by CLSM. A set of examples for all biofilm types (mono- and dual- species) is presented in Fig 9. A form of organization can be observed from the side views of the *Veillonella* mono-species biofilms and the *L. monocytogenes* and *Veillonella* dual-species biofilms. In the *Veillonella* mono-species biofilms, a clear stratification was observed with the dead cells (in green) in the upper layer of the biofilm and the living cells (in blue) located in the lower layer. In the dual-species biofilms, *L. monocytogenes* cells appear to occupy a larger portion of the upper biofilm while intermingling with dead *Veillonella* cells in the middle layer. In addition, *L. monocytogenes* cells seem to be in low concentration compared to *Veillonella* cells. Live *Veillonella* cells appear to be, once again, concentrated in the lower layer of the biofilms.

## Discussion

Various studies have shown that microorganisms can play a significant role in the survival of *L. monocytogenes* in food environments [22]. Many of these studies selected bacteria for co-culture with *L. monocytogenes* based on their presence in the same production compartment (e.g., cutting room) as the pathogen. Thus, these microorganisms were not necessarily previously co-isolated with *L. monocytogenes* (in a same sample), indicating that they may exist in other ecological niches than those occupied by the pathogen [7]. Fagerlund et al. have suggested that for an interaction to be considered relevant for the behavior of *L. monocytogenes*, the model should consist of bacteria found together with the pathogen in the food plant environment [7]. Using the biomarker analysis MaAsLin on 16S rRNA sequencing results in a previous study, we found that the presence of *L. monocytogenes* on contact surfaces of a cutting room of a swine slaughterhouse was associated with a higher relative abundance of the genus *Veillonella* [32]. Therefore, the aim of the present study was to recreate this interaction in the laboratory and thereby confirm the relevance of this interaction.

As a first step, *L. monocytogenes* (three strains) and *Veillonella* (two strains) were co-cultured in planktonic form under conditions favorable to the growth of both bacteria: in a rich medium (TSB), at 37°C, and in anaerobic conditions since *Veillonella* is strictly anaerobic. *L. monocytogenes* strains (V2CP2A (L1), V6PI6A (L2), and V3BO5A (L3)) were cultured alone and in co-culture with two *Veillonella* strains – ATCC17744 (V1) and ATCC17748 (V2). The measurement of absorbance, confirmed by enumeration of viable cell counts, showed significantly different maximal population densities (Nmax) between the individual and dual cultures. Indeed, a positive effect of the presence of *Veillonella* was observed with regards to the growth and/or to the survival of the pathogen. Regarding the monitoring of the absorbance of the different cultures, *L. monocytogenes* strains L1 and L3 growth when cultured alone ended at 48 hours, while the growth curves of several co-cultures continued over 72–96 hours (L1 + V2, L3 + V2) and/or reached a higher apex (L1 + V2, L3 + V1 and L3 + V2). L2 for its part, when grown alone and when cocultured with V1 exhibited an exponential phase extending over 48 hours (around 72 hours) whereas when cultivated with V2 its growth stopped at 48 hours. However, both co-culture curves (L2 + V1 and L2 + V2) showed a higher apex once again. Additionally, a greater number of cells as reflected by the higher turbidity of these cultures appeared to persist over time in the case of *L. monocytogenes* and *Veillonella* co-cultures and this for the three *L. monocytogenes* strains.

Regarding the viable counts of *L. monocytogenes* in mono- and co-cultures under planktonic conditions, similar growth patterns to those obtained for absorbance measurements were observed. Indeed, a significant increase in the number of live *L. monocytogenes* cells after six days of culture was observed for the L1, L2 and L3 strains in co-culture with the two *Veillonella* strains. The behavior of *L. monocytogenes* in positive relationships with food-associated bacteria has been characterized by a few studies in planktonic co-cultures [46–48]. Regarding positive interactions with the pathogen, Buchanan and Bagi studied *L. monocytogenes* in mono- and in co-culture with *Pseudomonas fluorescens* in BHI. At temperatures of 12°C and 19°C, slight increases (<1log cfu/mL) in the maximum population density attained by the pathogen were observed when grown in the presence of *P. fluorescens* [47]. These observations are in accordance with the results of our study. Indeed, we also observed an increase in the maximum density of *L. monocytogenes* populations. However, in our study several *L. monocytogenes/Veillonella* associations showed an increase of nearly or more than one log cfu per mL, depending on incubation times. Another study carried out by Guo et al. monitored the optical absorbance of *Ralstonia insidiosa* and *L. monocytogenes* grown individually or in co-culture in TSB and TSB/10 at 30°C [48]. The authors observed that the two bacteria showed a higher broth optical absorbance when co-cultured together than the sum of their single counterparts.

The results obtained under planktonic co-culture conditions in our study raise the hypothesis of possible metabolic cooperation between *V. dispar, V. atypica* and *L. monocytogenes*, in particular, the by-product of nutrients metabolized by the two *Veillonella* species could be utilized by *L. monocytogenes*. A study by Kara et al. showed that *Veillonella parvula* was able to catabolize lactate produced by *Streptococci* into shorter chain length acids, thereby indicating metabolic cooperation between the two bacteria, crucial in the establishment of oral biofilms [49]. In view of our results – and knowing that *Veillonella*’s primary carbon and energy source is lactate and that *L. monocytogenes* grown in glucose defined media is known to generate lactate, acetate, formate, ethanol, and carbon dioxide in anaerobic conditions [50] – the assumption of cooperation in terms of nutrient management between the two bacteria seems to be a promising avenue to explore.

To further investigate the relationship between the two bacteria, we studied the effect of *Veillonella* supernatant on planktonic cultures and on static biofilms of *L. monocytogenes*. First, viable counts of *L. monocytogenes* planktonic cultures consisting of different combinations of *L. monocytogenes* strains (L1, L2, or L3) and supernatants (SV1, SV2, SL1, SL2, or SL3) put in contact for specific time periods (24 hours or 48 hours) were realised at 37°C in TSB under aerobic conditions. The viable counts of the three *L. monocytogenes* cultures exposed to the supernatants of *Veillonella* were found to be significantly higher than the viable counts associated with the *L. monocytogenes* cultures exposed to their own supernatant. The three *L. monocytogenes* strains studied showed similar levels of growth enhancement. A 24-hour contact time between the supernatants and the different cultures of *L. monocytogenes* seems to be sufficient to increase the number of living cells of the pathogen to a greater extent than a contact time of 48 hours. This observation may be attributed to nutrient depletion and the accumulation of toxic products, which are inevitable in a non-renewed culture medium. This observation seems to be validated by the fact that high growth after 24 hours of contact appears to be particularly deleterious after 48 hours of contact. The supernatants harvested from the different *Veillonella* cultures after 48 (T2) and 96 (T4) hours of incubation showed the most significant increase in *L. monocytogenes* viable cell counts. This finding could be explained by the time required by *Veillonella* to produce the nutrient/compound/conditions that affect the growth of *L. monocytogenes*.

*L. monocytogenes* biofilms were grown under static conditions at 12°C in rich (TSB) and poor (TSB/10) media for 10 days. No biofilm growth was observed under poor nutrient conditions (data not shown). A similar result was obtained in a study conducted by Med da Silva Fernandes, although the incubation time observed in that study was shorter. At 7°C, despite the presence of *L. monocytogenes* in the culture medium, no biofilm formation was observed after a one-day incubation period. In our study, *L. monocytogenes* biofilms exposed to *Veillonella* supernatants (SV1 and SV2) showed significantly higher maximal population densities (Nmax) compared to biofilms exposed to *L. monocytogenes* supernatant in rich media. These differences were particularly noticeable on the seventh day of incubation. Similarly, several studies have reported the ability of certain bacteria to influence biofilm formation by *L. monocytogenes* [22,51]. The hypothesis of the enhanced survival rate of *L. monocytogenes* due to the presence of EPS has been raised with multiple bacteria [52–58,59,60]. For example, incubation of *L. monocytogenes* with *Enterococcus faecium* and *Enterococcus faecalis* resulted in a significant increase (2 log) in the count of *L. monocytogenes* in multi-species biofilms compared to the corresponding count in mono-species cultures [60]. However, this result could only be obtained at 25°C while competition for nutrients and the production and accumulation of toxic wastes seemed to limit the presence of *L. monocytogenes* in the biofilm at higher temperatures. According to the authors, this highlights the importance of temperature in the study of interactions within biofilms involving *L. monocytogenes* [60]. Interestingly, Carpentier et al. found a positive effect with a 0.5 to 1.0 log increase in *L. monocytogenes* biofilm CFU counts when co-cultured with *Kocuria varians*, *Staphylococcus capitis*, *Stenotrophomonas maltophilia*, and *Comamonas testosteroni* [59]. The authors did not find a link with the production of EPS, while the *C. testosteroni* filter-sterilized supernatant from a pure culture biofilm added to a pure culture of *L. monocytogenes* increased the number of pathogen cells adhering to stainless steel coupons [59]. However, in contrast with our study, the supernatant from the suspended cultures (not biofilms cultures) did not increase the *L. monocytogenes* CFU counts.

To further explore the effect of *V. dispar* supernatant on *L. monocytogenes* biofilms, L2 biofilms were grown under dynamic conditions and exposed to different supernatants. The L2 strain was selected among the three strains of the study due to its characteristics of interest in food microbiology. In a previous study, this strain was identified as belonging to serotype 1/2b and exhibited low biofilm production at 30°C and a moderate biofilm production at 12°C [32]. Many studies have investigated potential relationships between biofilm formation, phylogenetic division, and persistence in the environment of food production [61]. Several of them have suggested that strain-to-strain variations cannot explain why certain subtypes of *L. monocytogenes* persist in the food environment while others are only found sporadically [13,14]. Therefore, the resident background microbiota has been proposed to play a role in the protecting and sheltering of pathogens [13]. In our study, we investigated whether a *L. monocytogenes* strain characterized as a weak biofilm producer and belonging to lineage I – which has been identified as being overrepresented in human listeriosis cases although not predominant in the production environment could benefit from the presence of *V. dispar* supernatant products in a biofilm [62]. The *L. monocytogenes* biofilms exposed to the *V. dispar* supernatant for 24 hours showed a visible increase in the growth and attachment of *L. monocytogenes* cells (biovolumes 3.3 times larger than the biovolumes *L. monocytogenes* biofilms exposed to their own supernatants). The biofilms exposed only to the *V. dispar* supernatant during the last 20-hour (Condition C) flow appeared to have a cell density closer to those exposed to *L. monocytogenes* supernatant for 24 hours (Condition A) than to the biofilms exposed 24h to *Veillonella* supernatant (Condition B). This indicates a possible action of *V. dispar* supernatant compounds during the adherence phase of *L. monocytogenes*. Interestingly, when comparing the dead biomass and the live cell mass of the biofilms exposed to the different conditions, only the biovolumes of live cells of the *L. monocytogenes* biofilms exposed to the *V. dispar* supernatant for 24 hours (Condition B) showed statistically relevant differences compared to the biofilms exposed to the *L. monocytogenes* supernatant for 24 hours (Condition A). This result suggests that the action of the *V. dispar* supernatant is, at least partially, targeted at the level of the *L. monocytogenes* cells (live cell mass) instead of at the level of the *L. monocytogenes* associated biofilm matrix (dead biomass).

Recently, Mashima et al. showed that *Veillonella tobetsuensis* produces signaling molecules that promote the proliferation of *Streptococcus gordonii* during biofilm formation [34]. While the amount of the biofilm formed by *S. gordonii* alone decreased over time, the biofilms formed by both *S. gordonii* and *V. tobetsuensis* increased significantly [34]. The authors raised the hypothesis that *V. tobetsuensis* produces certain signals such as AI-2 that promote biofilm formation by *S. gordonii* [34]. However, they found that the AI-2-like molecule detected in the *Veillonella* supernatant inhibited the development of *S. g*ordonii biofilm, suggesting that the factor promoting the biofilm formation is likely to be extracellular molecules rather than AI-2 [34]. In light of these results, the hypothesis of a quorum sensing communication system between *V. dispar* and *L. monocytogenes* appears to be an interesting avenue to explore. However, very little is currently known regarding the occurrence of AI-2 activity in *Listeria* and its possible role in biofilm formation [63]. Other communication systems, such as acyl homoserine lactone (AHL), may also be involved. *L. monocytogenes* strains BN3 has recently shown to respond to AHL by expressing a virulence gene (*hlya*) and a biofilm formation gene (*srtA*) [64].

Regarding the appearance of biofilms built under dynamic conditions using poor medium (TSB/10), the organization in a knitted network with cells forming long chains as reported by Cherifi et al. was not observed in our study (CLSM; Biofilm assay using supernatants under dynamic conditions) [45]. Instead, a structure of homogeneous cellular multilayers associated with the formation of *L. monocytogenes* biofilms in rich medium was observed [45]. Some differences in the conditions and manipulations associated with the two studies may be involved. When the visualization of the mono- and dual-species biofilms of *Listeria monocytogenes* L2 and *Veillonella* V2 was performed using the *ViamGram*^TM^ Bacterial Gram Stain and Viability kit, no particular organization in the biofilm of *L. monocytogenes* under static conditions was observed by CLSM. However, the biofilm composed only of *V. dispar* cells showed a distinct two-layer organization pattern, with dead cells forming the upper layer of the biofilm and living cells located in the lower layer. This result can easily be explained by the anaerobic character of the *Veillonella* genus. In the dual-species biofilms, *Listeria monocytogenes* cells appear to occupy a larger portion of the upper biofilm while intermingling with dead *Veillonella* cells in the middle layer. Live *V. dispar* cells, once again, appear to be concentrated in the lower layer of the biofilms. It seems that in mixed biofilms, the proportion of *Veillonella* cells takes precedence over the number of *L. monocytogenes* cells. It may be that *Veillonella* provides shelter to *L. monocytogenes* cells, allowing some bacteria to persist without being exposed to the environment. This observation may be attributed to the laboratory conditions used in this assay. The presence of *V. dispar* cells (and not only the supernatant of its culture) could also be involved. Thus, the observed spatial organization is likely to be more complex in food production conditions.

In this study we were able to demonstrate, for the first time to our knowledge, that *Veillonella dispar* (ATCC 17748) and *Veillonella atypica* (ATCC 17744) have a positive impact on the growth and survival of *L. monocytogenes* in both planktonic cultures and biofilm under static and dynamic conditions. This ability of *V. atypica* and *V. dispar* appears to be mediated by compounds produced or made available by the bacterium since our experiments showed that contact with the supernatant of a *V. atypica* or *V. dispar* culture was sufficient to enhance the growth of *L. monocytogenes*. This increase appears to be primarily due to live cell mass, suggesting that *Veillonella (at least V. dispar)* acts at the cellular level rather than on the biofilm matrix of *L. monocytogenes*. We believe that our results represent a step toward a more complete food safety risk assessment. Indeed, *L. monocytogenes* should be considered part of a microbial consortium present in food processing environments that can affect the pathogen quantity on contact surfaces and subsequently on meat [22]. A better understanding of the microbial community associated with *L. monocytogenes* is essential for the development of improved strategies against this pathogen such as targeting bacteria that favor the presence of *L. monocytogenes* on food processing surfaces or, on the contrary, from a surface ecology perspective, identifying and using bacteria which are unfavourable to the presence of the pathogen.

## Supporting information

S1 Fig Inclusivity in global research questionnaire. (DOCX)
